# Combined Effect of Impregnation with an *Origanum vulgare* Infusion and Osmotic Treatment on the Shelf Life and Quality of Chilled Chicken Fillets

**DOI:** 10.3390/molecules26092727

**Published:** 2021-05-06

**Authors:** Maria C. Giannakourou, Stylianos Poulis, Spyridon J. Konteles, Akrivi Dipla, Vladimiros P. Lougovois, Vassiliki Kyrana, Charalampos Proestos, Vassilia J. Sinanoglou

**Affiliations:** 1 Department of Food Science and Technology, University of West Attica, Ag. Spyridonos 28, Egaleo, 12243 Athens, Greece; stylianospoulis@hotmail.com (S.P.); spycont@hotmail.com (S.J.K.); dipla.akrivi@gmail.com (A.D.); vloug@uniwa.gr (V.P.L.); vkyr@uniwa.gr (V.K.); 2 Laboratory of Food Chemistry, Department of Chemistry, National and Kapodistrian University of Athens Zografou, 15784 Athens, Greece; harpro@chem.uoa.gr

**Keywords:** chicken fillets, *Origanum vulgare*, osmotic treatment, hurdle technology, shelf life improvement

## Abstract

The scope of this work is the study of a combined process including a dipping step into an oregano (*Origanum vulgare* ssp. *hirtum*) infusion (OV) followed by osmotic treatment of chicken fillets at 15 °C. Chicken fillets were immersed in an osmotic solution consisting of 40% glycerol and 5% NaCl with (OV/OD) and without (OD) prior antioxidant enrichment in a hypotonic oregano solution. A comparative shelf life study of all the samples (untreated, OD and OV/OD treated) was then conducted at 4 °C in order to assess the impact of this process on the quality and shelf life of chilled chicken fillets. Microbial growth, lipid oxidation and color/texture changes were measured throughout the chilled storage period. Rates of microbial growth of pretreated fillets were significantly reduced, mainly as a result of water activity decrease (OD step). Rancidity development closely related to off odors and sensory rejection was greatly inhibited in treated fillets owing to both inhibitory factors (OD and OV), with water-soluble phenols (OV step) exhibiting the main antioxidant effect. Shelf life of treated chicken fillets exhibited a more than three-fold increase as compared to the untreated samples based on both chemical and microbial spoilage indices, maintaining a positive and pleasant sensory profile throughout the storage period examined.

## 1. Introduction

Production and consumption of chicken fillets has shown a significant increase in recent years, mainly due to the low cost compared to other meat products and the nutritional and sensory profile of such foods, which are a good source of proteins, vitamins and minerals [1,2], exhibiting low amounts of fat [3]. This kind of tissue, however, suffers from increased perishability, mainly attributed to its high water activity (a_w_), redox potential, elevated pH, aerobic conditions during storage, the presence of lipids and the high possibility of cross-contamination during handling and distribution. Besides confronting serious microbial spoilage, fresh poultry is readily contaminated by pathogenic bacteria such as *Salmonella* spp., *Campylobacter jejuni*, *Escherichia coli* O157:H7, *Listeria monocytogenes*, etc. [4]. All these factors and, most importantly, the growth of psychrophilic and psychrotrophic microorganisms, significantly reduces shelf life of chilled products to approximately 4–5 days [5]; another decisive factor in determining the shelf life of such perishable products that have also undergone pretreatment causing disruption of the integrity of muscle membranes is associated with lipid oxidation, even in cases where the fat content is as low as 0.5–1% [2]. These potential hazards constitute a challenge from a commercial point of view that has forced the food industry to seek processing strategies for extending the shelf life of fresh fillets. On the other hand, implementation of even minimal thermal treatments may lead to serious degradation of product appearance and structure. In this context, alternative methods of preservation besides cooling have been investigated, including freezing [6], high-pressure processing [7,8,9,10], modified atmosphere packaging [5,11,12,13], vacuum packaging [14,15], in-package cold plasma [16,17], irradiation [14], edible coatings [18,19], active packaging [20,21], etc. In many of the aforementioned published works, more than one technique were combined in order to maximize the benefits of the integrated process.

One preservation step frequently applied in the case of poultry products is related to the use of natural antioxidants incorporated within the meat tissue with a variety of methods, such as edible coatings, active packaging or by simply dipping in relevant infusions. As demonstrated in many published works, phytochemicals (e.g., flavonoids, other phenolic compounds) may act as reducing agents, free radical scavengers, metal chelators, and singlet oxygen quenchers [2], offering a wide range of benefits to the associated food item in terms of lipid oxidation inhibition. Extracts of plant origin are found to be more effective as antioxidants than their synthetic counterparts, protecting from free radical-mediated deteriorations and significantly delaying lipid oxidation. There are numerous works in recent literature demonstrating the antioxidant properties of several phytoextracts, proposing an alternative application of specific flowers/herbs as potential supplements in human nutrition [22]; another interesting aspect underlined in current research is the potential exploitation of phytochemicals and antioxidants present in plants, exhibiting several biological activities in improving human health [23,24,25,26]. One source of natural antioxidants is oregano (*Origanum vulgare*) frequently selected in the case of poultry not only for its well-established antioxidant activity, but also for its antimicrobial properties and its sensory affinity with chicken taste. In a study by Garavito et al. [27], oregano oil was added to the formulation of antimicrobial coating applied to fresh chicken breast fillets, and it was found that a significant extension of their shelf life (nine days vs. six days for untreated samples) was obtained. Boskovic et al. [28] studied the antioxidant stability of minced pork treated with thyme and oregano essential oils (EOs) and packaged under vacuum and modified atmosphere and observed reduced lipid oxidation after two weeks’ storage. Another combined process, namely, the effect of ethylenediaminetetraacetate (EDTA), oregano (*Origanum vulgare*) and thyme (*Thymus vulgaris*) oils followed by vacuum packaging under chilled storage of chicken breast fillets was examined, and the results showed that this treatment led to a significant reduction of all groups of microorganisms measured [15]. In another study [29], edible whey protein isolate (WPI) coatings with oregano or clove essential oils (EOs) incorporated as natural antimicrobials have been developed and tested on chicken breast fillets. The results confirmed the antimicrobial effect of oregano, since films containing oregano essential oils managed to limit most of the microorganisms tested below the recommended limits for consumption and were found to almost double the storage time of chicken breasts (from 6 to 13 days). Khanjari et al. [30] demonstrated that the combined effect of an N,O-carboxymethyl chitosan (NOCC) and 1% oregano essential oil (OEO) dip was very effective in controlling growth of *Listeria monocytogenes* on chicken breast fillets as compared to controls or samples only treated with oregano essential oils. From an economic perspective, using dried ground leaves of oregano could be advantageous in an effort to reduce the necessary processing and the high costs of oil extraction as long as the antimicrobial properties of such dry matter could be established. Sobczyk et al. [31] showed that although the content of active compounds thymol and carvacrol was lower in ground leaves as compared to the essential oil, there was an evident antibacterial activity of this oregano form against gram-positive and gram-negative bacteria, confirming the beneficial effect of the bioactive components of this plant. Detailed determination of bioactive compounds present in dried oregano leaves is provided in [32] where authors investigated alternative extraction methods. In another study [33], the total phenolic content, antioxidant activity and antibacterial properties of water (hot and cold) and ethanolic extracts of air-dried oregano plant parts were measured and the results confirmed the beneficial effect of such extracts, especially as antioxidant agents. Regarding dried *Origanum vulgare* leaves, previous studies of our research team reported identification of their phenolic substances by high-performance liquid chromatography (HPLC) coupled with multi-wavelength ultraviolet-visible (UV-vis) spectroscopy as well as NMR metabolomics and spectrophotometric studies (Folin–Ciocalteu, FRAP, ABTS) to characterize their infusions and decoctions [34,35]. According to Proestos et al. [35], the most abundant bioactive phenolics identified in oregano leaves were caffeic and ferulic acids as well as the flavonoids rutin, quercetin and catechin.

Osmotic dehydration (OD), a well-established mild non-thermal technique, includes product immersion in a hypertonic solution of different solutes, aiming at targeted mass transfer through a selectively permeable cell membrane. The result is simultaneous dehydration and tissue enrichment with preselected desirable compounds. Therefore, depending on the desired attributes of the final processed product, it is possible to optimize the main process parameters based on an appropriate experimental design followed by a corresponding kinetic study. One of the most important issues in OD implementation is related to the selection of the osmotic agent depending on its water activity-lowering force, effect on the final product’s organoleptic profile and safety, as well as economical aspects. In this context, a tertiary solution containing sodium chloride and glycerol (an effective humectant and plasticizer that enhances product texture) is often used in muscle foods [36,37,38,39]. Application of OD is mainly studied in plant tissues, with examples on meat products being rather rare. A range of authors [40,41] studied the effect of OD process parameters on mass transfer phenomena and microbiological stability of chicken breasts applying, among other things, the response surface methodology (RSM) for modeling and statistical evaluation purposes. Other research teams [29,42,43,44] have systematically studied the effect of binary and tertiary osmotic solutions on mass transfer kinetics of beef meat. In 2018, Andreou et al. [10] studied the combinatory effect of osmotic treatment and high-pressure processing on chicken breast samples, concluding that, based on microbial growth criteria and sensory attributes, an extension of shelf life at 5 °C was obtained, close to 9, 16, 25 days for OD, HP and OD-HP samples, compared to six days for non-treated samples. Schmidt et al. [45] investigated the applicability of different empirical models to describe the diffusion occurring during the OD of chicken breast cuts immersed in salt solutions of different concentrations. In all cases when a safe product is required, OD should always be combined with another preservation step since OD by itself cannot ensure product stability, mainly due to elevated water activity.

Bearing in mind current consumer needs for fresh or minimally processed, microbiologically safe and healthy foods of high quality, a concept called hurdle technology has been proposed. This approach is based on the careful combination of traditional and novel preservation methods called ‘hurdles’ in order to inhibit growth of microorganisms and other deterioration reactions [46,47,48,49,50,51,52,53,54]. In this context, the possibility of combining chilled storage with other techniques in the context of the ‘hurdle technology’ like the direct addition of chemical preservatives or, preferably, natural antimicrobial compounds is extensively studied as an effective way to further prolong the shelf life of animal tissue [29,55,56,57]. However, there are limited studies in current literature that have investigated the benefits of applying an antioxidant impregnation step followed by an osmotic dehydration process; such a complementary preservation technique has been implemented in the case of aquatic products [39,58,59,60].

The aim of this research is the application of a combined technique, including the enrichment of chicken fillets’ tissue with bioactive compounds, followed by an OD step, mainly aiming at water activity reduction. The methodology is based on the measurement of microbiological, chemical and sensory changes of air-packaged, refrigerated chicken fillets pretreated in a hypertonic osmotic solution with and without prior immersion in an oregano infusion. The ultimate goal was to determine the shelf life improvement obtained during the subsequent chilled storage. This study represents an example of hurdle technology implementation by coupling two mild preservation steps, namely, impregnation with a natural extract so as to enhance the antioxidant and antimicrobial activity of the product and a water activity reduction process (mainly through water loss from the interior of chicken fillets).

## 2. Results and Discussion

### 2.1. Bioactive Compound Impregnation Using Origanum vulgare Infusion and Osmotic Dehydration (OV/OD and OD)

#### 2.1.1. Bioactive Compound Impregnation Using *Origanum vulgare* Infusion and Osmotic Dehydration (OV/OD and OD)—Mass Transfer and Impregnation Phenomena

**Figure 1:**
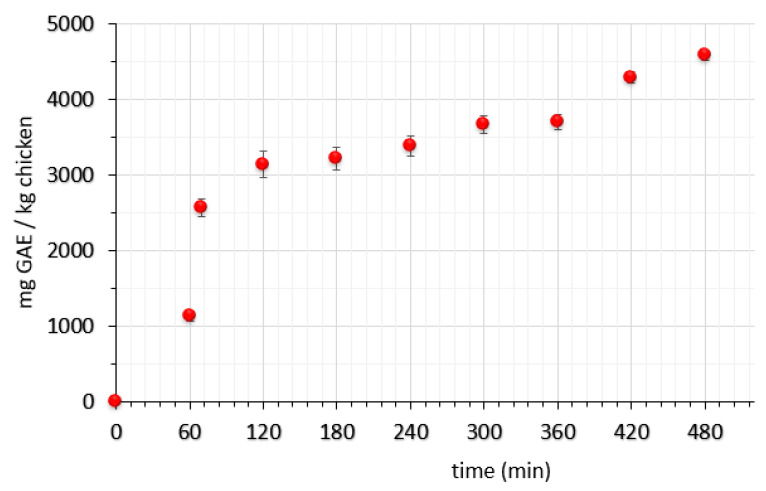
Impregnation of phenolics into chicken muscle, expressed as mg GAE/kg of chicken muscle (OV/OD procedure). Error bars represent the ± standard deviation of multiple measurements.

The total phenolic content of oregano (*Origanum vulgare*) infusion measured by the Folin–Ciocalteu method was found to be 3260.40 ± 32.53 mg GAE/L. Fotakis et al. [34] presented detailed implementation of NMR metabolomics and spectrophotometric studies (Folin–Ciocalteu, FRAP, ABTS) to infusions of *Origanum vulgare*, where a total phenolic content of 215.24 ± 7.92 (mg of GAE/100 mL), antiradical activity of 132.16 ± 0.36 (mg of Trolox equivalent)/100 mL) and antioxidant activity of 82.74 ± 5.06 (mg FeSO_4_∙7H_2_O/100 mL) are reported. As far as specific types of phenolics and flavonoids present in dry oregano leaves are concerned, in 2013, Proestos et al. [35] identified and quantified these compounds using an HPLC system coupled with a multi-wavelength ultraviolet-visible (UV-vis) spectroscope, reporting caffeic and ferulic acids as the main phenolic compounds and rutin, quercetin and (+)-catechin as the principal flavonoids in these plant extracts. In our case, immersion of chicken fillets into an *Origanum vulgare* infusion led to a significant enrichment of meat flesh with bioactive compounds as depicted in Figure 1. During the first stage of the dipping treatment (OV) with a hypotonic solution, penetration was rather rapid, but a sharp increase was observed at the point where the pre-weighed osmotic solutes were added and the solution became hypertonic. During the second stage of osmotic treatment, phenolic content was found to be almost stable, an observation that confirms that the penetrated bioactive compounds were well-bound to the flesh and did not show a back diffusion mass transfer, a finding that agrees with [39,60] previous studies. This may be attributed to two synergistic phenomena that hinder leakage of phenolic compounds: formation of a protective layer on the sample’s surface due to the concentration of the main osmotic agent, namely, glycerol [61], and gradual diffusion of low-molecular-weight compounds towards the center of chicken fillets due to the increase of osmotic pressure gradient through the semipermeable cellular membrane. The overall 8-h dipping in the 40% glycerol solution led to a final content of approximately 4.6 mg GAE/g of chicken fillets (4600 ppm). This result could be considered an efficient antioxidant concentration for the chicken fillet muscle since it does not contain any antioxidants as a raw animal tissue.

**Figure 2:**
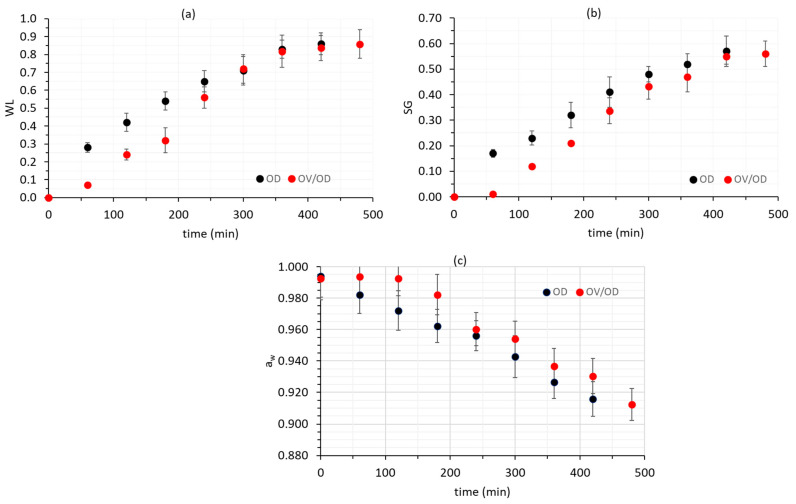
(**a**) Water loss (WL), (**b**) solid gain (SG) and (**c**) a_w_ decrease of chicken fillets samples after OD and OV/OD at 40% glycerol concentration of the osmotic solution. Error bars represent the ±standard deviation of multiple measurements.

Regarding mass transfer phenomena, the osmotic treatment by itself, as well as the combined process (OV/OD), caused a significant (*p <* 0.05) water loss (WL) from chicken muscle, combined with a significant (*p <* 0.05) solid gain (SG). Moreover, a_w_ changes showed very similar trends in both alternative procedures taking into account that in the first 60 min of OV impregnation, virtually no mass transfer was recorded (Figure 2a–c). Samples reached a final a_w_ value of approximately 0.9150, which is a satisfactory decrease for shelf life extension purposes.

#### 2.1.2. Color Change

**Figure 3:**
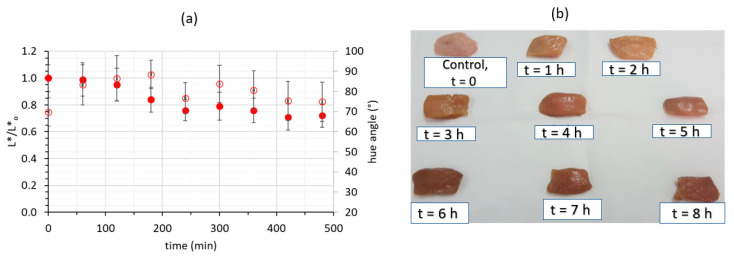
(**a**) Color changes expressed as L*/Lo* (full circles) and hue angle (open circles) during the combined procedure (OV/OD). Error bars represent the ±standard deviation of multiple measurements. (**b**) Representative illustrations of chicken fillet samples during the 8-h OV/OD process.

Color expressed as the relative loss of the initial lightness (L*/Lo*) showed no significant (*p* < 0.05) change during neither of the procedures (OV/OD and OD) (Figure 3). When samples were initially immersed into the OV infusion, they obtained a more yellowish color as is mostly depicted by the hue angle due to the effect of the colored *Origanum vulgare* infusion. From Figure 3, it can be observed that hue values of the raw material are in the reddish area, whereas during impregnation of samples with an OV infusion, values tend to 90°, signaling a more yellowish appearance, attributed possibly to colored phenolic compounds introduced within the chicken muscle. After 60 min of OV dipping and after rendering the solution hypertonic (OV/OD) with the addition of glycerol, lightness was slightly reduced and the hue angle was stabilized near the value of 80°, changes which are responsible for the final light, orange-brownish color of chicken samples. Regarding the one-step OD procedure, neither lightness nor hue angle exhibited significant differences during the 8-h dipping process (*p* < 0.05).

### 2.2. Stability Study and Quality Changes during Chilled Storage

#### 2.2.1. Microbial Growth of Spoilage Bacteria

Before its use in the OV/OD procedure, the presence of any initial microbial load of the oregano infusion was assessed via the total viable count, and it was found below the detection limit. The growth curves of the tested spoilage bacteria (total viable count and psychrotrophic bacteria) in chicken samples (untreated, OD and OV/OD) stored at 4 °C were fitted to the Baranyi growth model ([62], DMfit, available at http://www.combase.cc/index.php/en/ (accessed on 1 March 2021)), and the growth kinetic parameters at each processing condition were calculated (R^2^ = in the range between 0.901–0.999). No lag phase was observed in the growth of any of the measured bacteria. The microbial growth rate (k, days^−1^), i.e., the rate at the linear phase of the measured microorganisms, the initial population (N_o_ in log CFU/g) and the final population (N_max_ in log CFU/g) are presented in Table 1.

The initial count of all bacterial groups tested both for OD and OV/OD-treated samples was lower (*p* < 0.05) than that of the untreated counterparts, and that could be attributed to the aforementioned treatments. Additionally, the growth rates (k, days^−1^) at 4° C were significantly lower in the OD and OV/OD chicken samples compared to the raw ones (Table 1), and this is the cause for the faster deterioration of the raw samples in comparison to their treated counterparts.

**Figure 4:**
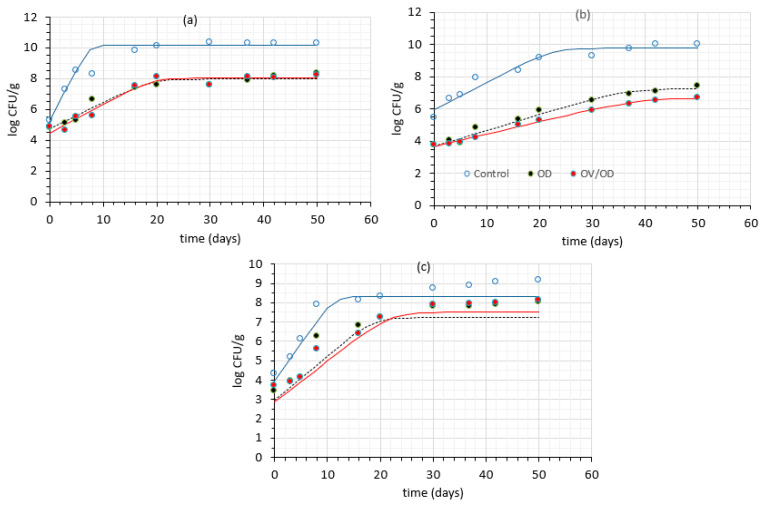
Microbial growth of (**a**) total viable count, (**b**) psychrotrophic bacteria and (**c**) LAB for all samples stored at 4 °C.

In Figure 4, experimental data and the corresponding fitted growth curves are depicted, from which it can be observed that in the untreated samples, the logarithmic growth phase was always much shorter compared to that of pretreated samples. Untreated samples reached a population of 10^8^ CFU/g after almost five days of chilled storage, which signals end of acceptability of chicken tissue since it is accompanied by an unpleasant odor. The limit of 7–8 logCFU/g is often used for sensory rejection of chicken muscle since when the product exceeds 8 log CFU/g, it is considered irreversibly spoilt [10,63,64,65,66].

On the other hand, from the results illustrated in Figure 4b, it can be deduced that psychrotrophic bacteria reached a population of more than 10^9^ CFU/g within 20 days in the untreated samples, while at the same time the population of the treated samples (OD and OV/OD) was approximately 3.5 log cycles lower. Psychrotrophic bacteria are the dominant spoilage microorganisms in aerobically packed chicken samples, which agrees with [10,67,68]. It is clear that lowering a_w_ (OD step) to a level of < 0.95 acts as an effective barrier to psychrotrophic species growth [69,70], leading to a substantial reduction of the initial load and a significant delay of microorganisms’ growth.

Regarding lactic acid bacteria also analyzed in chicken samples, the results were coherent with those of total viable counts and psychrotrophic population. More specifically, a rather rapid growth was observed in untreated samples, with microbial load starting from 4 log CFU/g at day 0 and reaching final loads of 7.9 log CFU/g after eight days of refrigerated storage. The OD step resulted in a significant delay of LAB growth, with the final load measured not exceeding 6.5 log CFU/g during the eight-day chilled storage. These results are in agreement with [71,72].

The obtained results (Figure 4 and Table 1) demonstrate the beneficial impact of both a_w_ lowering (through the OD procedure) and the synergistic effect of the bioactive compounds’ impregnation (OV/OD process) since the limit of 8 log CFU/g was not exceeded even after 50 days of chilled storage. Nonetheless, no statistically significant difference in the antimicrobial activity was found between the two alternative procedures (OD and OV/OD), as was also confirmed by ANOVA statistics (Table 1). Similarly to our previous observations [39], dipping into an oregano infusion (OV step) did not affect significantly the growth of any of the investigated species in osmosed chicken muscle (*p* > 0.05). The results obtained in this study showed that the OD step and the subsequent a_w_ decrease obtained had a more pronounced effect on microbial growth, with bioactive compounds of oregano introduced within chicken flesh having a milder non-significant antimicrobial impact.

In general, there is a large body of scientific data that supports the antimicrobial activity of polyphenolic components, including poultry meat [71,73,74,75,76]. More particularly, oregano extracts were used in certain recent works for inhibiting microbial proliferation in meat flesh. Pavelková et al. [15] applied oregano, thyme oil and EDTA in combination with vacuum packaging and observed a significant effect on microbial growth on chicken breast fillets. The effectiveness of plant extracts containing rosemary and oregano was studied by Sood et al. [77] as a means to extend the shelf life of bison strip loin steaks in terms of color stability and consumer acceptability. Carvacrol and thymol which are identified as the main compounds of *Origanum vulgare* extracts are associated with the observed antimicrobial activity of this spice [78]. The antimicrobial activities of phenolic compounds may include several mechanisms, such as degradation of cell walls, disruption of the cytoplasmic membrane, leakage of cellular components, alterations of fatty acids and phospholipid compounds, impact on the synthesis of DNA and RNA, etc. [79].

One should notice, however, that a common point in the majority of those studies was that the extracts contained essential oils, which very often led to food with unusual and often undesirable change of organoleptic characteristics (flavor, taste).

#### 2.2.2. Lipid Oxidation

**Figure 5:**
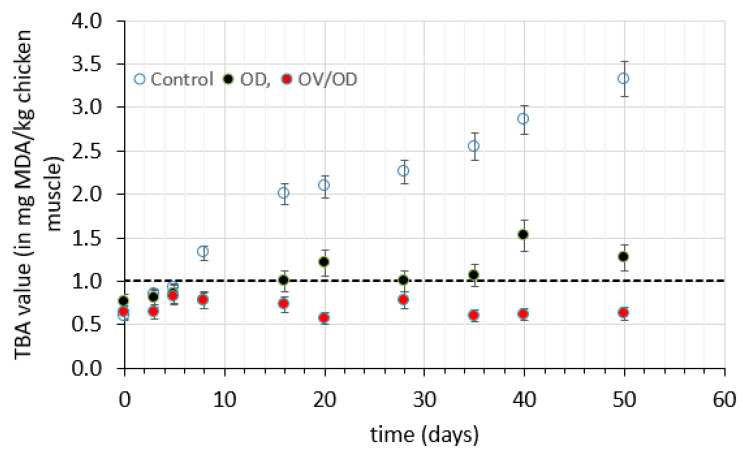
Lipid oxidation of chicken fillets (expressed as concentration of malondialdehyde, mg MDA/kg of chicken muscle) during storage at 4 °C.

Besides microbial spoilage, chemical deterioration and, most particularly, lipid oxidation is a predominant factor limiting the shelf life of muscle food [71]. Content of malondialdehyde in foods measured by the amount of thiobarbituric acid (TBA) is often used as an index of secondary oxidation products. In our study, the initial malondialdehyde (MDA) content was in the range between 0.6–0.8 mg MDA/kg of chicken flesh, which is in agreement with [71,80,81]. The TBA value showed a significant increase (*p <* 0.05) in untreated samples exceeding the acceptability limit of 1 mg MDA/kg sample within six days (Figure 5). This limit is considered to be strongly associated to rancidity sensorially perceived by consumers and is often used as the rejection criterion set for shelf life determination in raw meats [81,82,83]. Moreover, TBA values of OD samples increased significantly (*p* < 0.05), exceeding the limit of 1 mg MDA/kg sample after 20 days and reached a value of 1.27 mg MDA/kg after 50 days. Interestingly, TBA values of OV/OD samples showed insignificant variation, without exceeding the value of 0.63 mg MDA/kg even after 50 days of chilled storage. Regarding the above results, it is evident that the synergistic effect of impregnation with bioactive oregano compounds with the a_w_ lowering obtained by the OD step suspended the rate of lipid oxidation, which constitutes a major factor of spoilage of chicken muscle.

In the recent literature, there are several studies investigating the protective role of different types of extracts rich in bioactive compounds on the oxidation of chicken tissue. Aziz et al. [76] provided a complete overview of the use of natural antimicrobials and antioxidants for improving shelf life of meat products. Antimicrobial and antioxidant effects of different spice extracts (including *Origanum vulgare*) in raw chicken meat during chilled storage were studied in [71], and their beneficial impact on product shelf life was confirmed. This result, which is in agreement with our findings, may be attributed to the antioxidant activity of phenolic compounds introduced in the impregnation step. These bioactive compounds of high redox potential may act as reducing agents, hydrogen donors and singlet oxygen quenchers [84]. As detailed by the authors, this beneficial activity may be justified by the presence of the hydroxyl group linked to the aromatic ring which is capable of donating hydrogen atoms with electrons. Raeisi et al. [82] investigated fortification of chicken nuggets with encapsulated fish oil combined with garlic essential oil for shelf life extension through lipid oxidation delay during cold storage.

#### 2.2.3. Color and Texture Change

**Figure 6:**
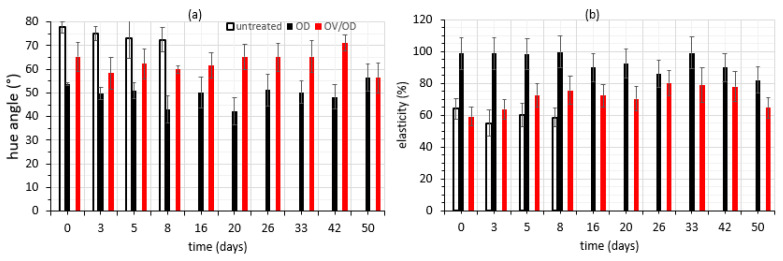
(**a**) Color changes expressed as hue angle and (**b**) elasticity alterations of all samples during storage at 4 °C. Error bars represent the ±standard deviation of multiple measurements.

Storage at 4 °C affected color parameters of untreated and pretreated chicken fillets only to a slight extent, with L* values of all series of samples retaining their initial level; however, lightness of OV/OD samples was lower than that of OD samples and significantly lower than that of the untreated ones, as expected from the effect of the dipping process into the colored oregano infusion (OV step) (data not shown). The above results regarding L* value decrease are in agreement with those reported in the literature [10], as the osmotic treatment leads to a modification of the surface layer composition of chicken fillets via glycerol deposition. Redness (a*) of OD and OV/OD samples was significantly higher than that of the untreated ones, whereas yellowness (b*) showed no significant (*p* > 0.05) variations among samples as a consequence of the osmotic treatment. Results on both a* and b* values of OD and OV/OD samples revealed their stabilization throughout the storage period (data not shown). As far as the hue angle is concerned, OV/OD chicken samples exhibited good retention of their yellowish to reddish color (values up to 65°) obtained after OV impregnation, whereas OD samples retained a more yellowish hue (with lower values, close to 50°). All the treated samples exhibited a brighter yellow/white color, with lower hue values than the untreated samples that obtained values close to 75° (Figure 6a). In this research, color changes of chicken muscle during storage showed that the phenolics impregnated into the chicken tissue, protected more effectively against color alterations, probably owing to the proposed combined process.

As far as texture changes are concerned, elasticity of samples seemed to be abruptly increased immediately after the OD treatment, as expected due to the mass transfer flows occurring (Figure 6b). The application of an initial impregnation OV step seemed to lead to a softer increase of sample elasticity compared to the untreated ones; in any case, all categories of samples exhibited good retention of their initial elasticity values during the whole storage period examined. Literature data also mention that the osmotic solution composition significantly affects the textural parameters of osmotically processed meat [85].

#### 2.2.4. Sensory Evaluation

**Figure 7:**
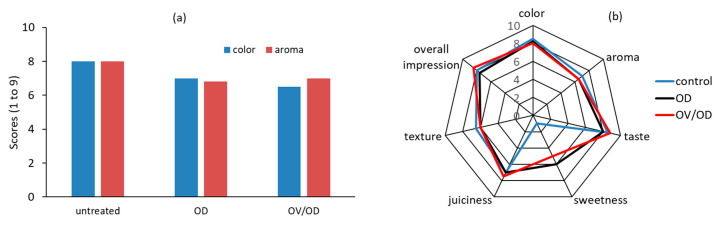
(**a**) Sensory evaluation of color and aroma of raw samples (at time zero of storage) and (**b**) sensory evaluation of cooked chicken fillets for all categories tested (untreated, OD and OV/OD).

The purpose of the sensory testing was mainly to evaluate consumer acceptability towards the modified chicken products immediately after the combined treatment and prior to the chilled storage. Therefore, the sensory characteristics (appearance, aroma) of raw untreated, OD and OV/OD chicken samples were assessed at time zero of chilled storage, with samples impregnated with an OV infusion exhibiting lower scores in appearance (Figure 7a) as expected from the dark color induced by the colored infusion. However, when cooked samples were tested (at time zero of storage time), both series of OD and OV/OD samples were highly appreciated, with impregnated samples receiving significantly higher scores (Figure 7b). As far as chilled storage is concerned, sensory rejection of raw, untreated chicken samples attributed to detectable off-flavors was directly correlated to both microbiological and chemical (rancidity development) spoilage, and a maximum shelf life of six days was recorded. Regarding OD samples, osmotic pretreatment maintained the sensory attributes of raw chicken samples until approximately 20 days of cold storage, a period that coincides with microbial growth over 8 log CFU and lipid oxidation limit exceeding the value of 1 mg MDA/kg chicken muscle. On the other hand, raw oregano-pretreated samples (OV/OD) maintained an acceptable sensory profile throughout the testing period of approximately 50 days, without testers being able to distinguish any unpleasant odor until the end of the experimental period. It should also be noticed that only a mild pleasant odor characteristic of the oregano spice was detected during cold chicken fillet (OV/OD) storage.

#### 2.2.5. Shelf Life Determination

Regarding shelf life determination, the determining criteria set were either based on microbial growth (the limit of 10^8^ CFU/g of microbial count in accordance with [10,63,64,65] or lipid oxidation development, assuming a limit of acceptability at the end of the shelf life of poultry products at 1 mg MDA/kg chicken flesh [84,85,86]).

Based on the above criteria, the shelf life of the different chicken samples could be estimated using the following equation (Equation (1)):

(1) $$\log\left(\frac{N_{t}}{N_{o}}\right)=kt\Rightarrow t_{SL}=\frac{\log N_{t}-\log N_{o}}{k}$$

where *N_t_* and *N_o_* are the final (limit of acceptability) and the initial microbial population and *k* is the relative growth rate of TVC (Table 1). Therefore, their shelf life was found to be approximately ten days for the untreated samples and more than 30 days for the OD and OV/OD treated ones.

If rancidity development is the crucial factor for shelf life evaluation and is based on the limit of acceptability of 1 mg MDA/kg chicken flesh, one could graphically estimate the shelf life of the different pretreated samples, namely, six days for the untreated samples, 15 days for the OD-treated ones and a much longer time period (not perceived from the plot) for the OV/OD ones. Therefore, it can be concluded that at the particular storage temperature (4 °C) studied, chicken samples tend to deteriorate more rapidly in terms of lipid oxidation (associated with development of off-odors, also perceived from the relative sensory evaluation performed during the storage period), with microbial growth showing slightly lower rates. This observation is in agreement with [10], where it is also demonstrated that at different temperatures the prevailing degrading mechanism may be different. In this published work, it is shown that at higher, abusive temperatures, microbial growth is the limiting factor, whereas at lower temperatures, sensory rejection caused by rancid development might be the cause of rejection.

## 3. Materials and Methods

### 3.1. Sample Preparation

Chicken breast fillets were purchased from a well-known Greek meat producer. The samples were cut into rectangular slices (3 × 3 × 1 cm^3^, 13 ± 2 g) in a laminar flow hood. Regarding the water-soluble bioactive compounds impregnation step, an oregano infusion (*Origanum vulgare* ssp. *hirtum*) commonly known as ‘Greek oregano’ from the cultivar ‘Greek Kaliteri’ was prepared by dissolving under continuous agitation a pre-weighed quantity of dehydrated oregano leaves in distilled water at 80 °C for 15 min in a ratio of about 1:20. The solution was microbiologically analyzed by plate counting (PCA/30 °C/72 h) and the total viable count (TVC) was below the detection limit of the method. The mixture was then strained off, filtered, and this solution also served as the basis for the OD solution (step 2) after the impregnation procedure was completed. The osmotic solution (step 2) was prepared by dissolving pre-weighed quantities of food grade glycerol (Honeywell, Riedel-de-Haen, Charlotte, NC, USA) at a concentration of 40% and 5% NaCl (OD conditions were based on preliminary experiments and the results published in [34,58]) either in distilled water or in the oregano infusion (step 1). Glycerol is a sugar alcohol (E 422, group I) of low molecular weight exhibiting a mild sweet taste, recognized as a non-toxic and safe additive (Regulation (EC) No. 1333/2008). The use of glycerol in foods is not unusual and is legally permitted by the EU legislation in several food groups like cocoa and chocolate products, flavored fermented milk products including heat-treated products, fillings of stuffed pasta and, most importantly, for non-heat-treated meat products (food category 8.3.1 according to Regulation (EC) No. 1333/2008). In this tertiary hypertonic solution, NaCl was also used in order to improve the mass transfer of the process and counterbalance the possible mild sensory modification observed due to glycerol application.

### 3.2. Bioactive Compounds Impregnation and Osmotic Dehydration

Three series of chicken fillet samples were prepared: untreated, fillets directly submitted to the osmotic dehydration procedure (OD) and fillets initially immersed into a hypotonic oregano infusion for 60 min in a food/infusion ratio of 1:2 before adding the pre-weighed osmotic agents of glycerol and NaCl (OV/OD) (with a final concentration of 40%). This sequential two-step procedure instead of a simultaneous immersion process was selected based on previous observations [60,86] and on our own preliminary experiments, where it was verified that a one-step procedure does not lead to a significant antioxidant enrichment of chicken muscle.

For OD and OV/OD series, the osmotic dehydration process was performed at 15 °C for a duration of up to 420 min with a food/solution ratio of 1:4. To perform the osmotic treatment, coded beakers filled with pre-weighed osmotic solutions were placed in an incubator (POL-EKO-APARATURA SP.J., type ST 1 B SMART) at 15 ± 1 °C. Pre-weighed chicken samples were kept immersed within the hypertonic solution by means of a grid. At predetermined timepoints, one beaker was removed, the samples were gently blotted with a tissue paper to remove the excess solution and then weighed.

Mass transfer kinetics were evaluated by calculating WL (water loss) and SG (solid gain). The measurements were performed in triplicate and the average values were taken. Moisture content was measured by sample drying at 70 °C under vacuum (Heraeus Instruments Vacutherm) for 24 h.

(2) $$WL=\frac{\left(M_{0}-m_{0}\right)-\left(M-m\right)}{m_{0}}\;(g\;\mathrm{of}\;\mathrm{water}/g\;i.d.m.)$$

(3) $$SG=\frac{m-m_{0}}{m_{0}}\;(g\;\mathrm{of}\;t.s./g\;i.d.m.)$$

where *M*_0_ is the initial mass of fresh material before the osmotic treatment, *M* is the mass of chicken samples after time t of the osmotic treatment, *m* is the dry mass of chicken after time t of the osmotic treatment and *m*_0_ is the dry mass of ‘fresh material.’ Abbreviation i.d.m. stands for ‘initial dry matter,’ t.s.—for ‘total solids.’

Water activity was determined using an a_w_-meter (AquaLab Dew Point Water Activity Meter 4TE), and °Brix of the osmotic solution was measured with a hand-held refractometer (Atago, Master refractometer, Japan).

### 3.3. Total Phenolic Content (TPC) Determination during Immersion in an Oregano Infusion

The total phenolic content of chicken fillets during impregnation and the subsequent osmotic step was measured indirectly by assessing the TPC of the hypotonic/hypertonic solutions according to the modified micromethod of the Folin–Ciocalteu’s assay as described in [87]. The TPC was expressed as mg of gallic acid equivalents (GAE) per g of chicken flesh and all measurements were performed in triplicate.

### 3.4. Shelf Life Kinetic Study

The main purpose was to design and perform a comparative stability study of all series of samples (untreated, OD and OV/OD) in order to compare deterioration of their quality during chilled storage. The purpose was to evaluate their shelf life and assess the effect of the different treatments imposed. The samples were stored at controlled isothermal conditions of 4 °C in a low-temperature incubator (POL-EKO-APARATURA SP.J., type ST 1 B SMART) after being air-packaged and thermally sealed in a laminate PET/Al/LDPE film. At predetermined time intervals, namely, at time 0, days 3, 5, 8, 16, 20, 30, 37, 42 and 50 of isothermal cold storage, the samples were withdrawn from the incubator and analyzed for a number of quality indices as detailed in the following sections. The approximately two-month analysis, although considered too extended for control samples, was deemed necessary, as some indices (e.g., lipid oxidation, psychrotrophic bacteria growth and off-odor development) remained at remarkably low levels in the case of their treated counterparts (OV/OD samples).

#### 3.4.1. Microbiological Analysis

For microbiological analyses, 10 g of representative chicken flesh were homogenized with 90 mL of sterilized Ringer solution (Merck, Darmstadt, Germany) in a sterile bag for 60 s using a Stomacher (BagMixer^®^, Interscience, Saint-Nom-la-Bretèche, France), and then ten-fold serial dilutions were made with the same solution. Aliquots of each dilution (1 mL) were added to Petri dishes, to which an appropriated agar medium was then poured. The enumeration of total viable counts (TVC) and lactic acid bacteria (LAB) was made in plate count agar (PCA, Merck, Darmstadt, Germany) plates incubated at 30 °C for 72 h and in the double-layered de Man, Rogosa and Sharpe Agar (MRS, Merck, Darmstadt, Germany) incubated at 37 °C for 96 h, respectively. Regarding the enumeration of psychrophiles, 0.1 mL of each serial dilution was spread-plated on the plate count agar and incubated at 8 °C for 120 h.

Microbial growth modeling was performed using the Baranyi growth model [62] by fitting curves using the DMFit program. Different kinetic parameters, namely, the rate (k) of the microbial growth and the final microbial population predicted (N_max_) were estimated at the processing conditions investigated.

#### 3.4.2. Valuation of Lipid Oxidation

Oxidation in chicken flesh was evaluated with the TBARS method, which is preferably applied to foods of animal origin since it is well-associated with sensory perception [88]. Lipid oxidation was estimated by the thiobarbituric acid assay according to the extraction method described in [89]. The absorbance was measured at 530 nm using a digital spectrophotometer (Hitachi U-3210; Hitachi, Ltd., Tokyo, Japan). Concentrations of thiobarbituric acid reactive substances (TBARS) were calculated from a standard curve prepared using 1,1,3,3-tetraethoxypropane and expressed as mg malondialdehyde (MDA) per kg of chicken flesh. Each measurement was carried in triplicate and the average values along with their standard deviations were calculated.

#### 3.4.3. Sensory Evaluation

At time zero of chilled storage, the sensory attributes of both raw and roasted chicken fillets from all the categories, namely, control, OD and OV/OD, were evaluated by a 15-member experienced and trained sensory panel. Testers were recruited and followed a training session on the basic organoleptic properties regarding the particular product. Appearance and odor of raw samples and color, flavor, taste, texture, juiciness, sweetness and overall impression/acceptability of their roasted counterparts were evaluated. A similar study is presented in [10,67], where sensory evaluation was performed by a seven-member and five-member trained sensory panel, respectively, on uncooked and cooked chicken breast fillet samples. In [90,91], a panel of seven experienced judges evaluated organoleptic properties of cooked chicken burgers and chicken breast samples, respectively. Raeisi et al. [82] used a sensory panel that comprised ten experienced subjects to evaluate sensory attributes of deep-fried chicken nugget samples. In a study by Garavito et al. [27], cooked chicken breast fillets were evaluated by eight semi-trained panelists.

In our work, roasting took place in a domestic oven at 200 °C for 15 min, and all the samples were wrapped in thin aluminum foil to avoid extensive dehydration. After their thermal treatment, the samples were left to cool down to approximately 25 °C before being coded and evaluated by the panelists. Ratings were assigned separately to each parameter on a 1-to-9 hedonic scale (where 9 = like extremely and 1 = dislike extremely). A sensory score of 5 was taken as the average score for minimum acceptability. During the subsequent chilled storage of all the samples, no systematic sensory evaluation was performed; appearance and odor of all the raw samples was merely recorded in order to gain some information on the potential correlation of sensory rejection with microbial growth/lipid oxidation measured throughout this period.

#### 3.4.4. Color and Texture Measurements

Color of chicken samples was instrumentally assessed with a tristimulus chromatometer (model CR-400, Minolta, Tokyo, Japan) calibrated with a white standard plate (L*: 97.83, a*: −0.45, b*: +1.88), where CIELAB color scales were used, with coordinates (L*, a*, b*) being directly read from the chromatometer. Bearing in mind the light white-to-yellowish hue of chicken muscle, lightness (described by the L* parameter) and the hue angle (h* = tan−1(b∗/a∗)) were chosen as the most representative color factors to be recorded. Hue angle is a very useful attribute since it describes the hue of the sample, and therefore it is found to represent adequately consumer perception of color. Actually, hue angle value defines the difference of a particular color vs. the grey color with the same lightness [92].

Texture measurements were performed with a texture analyzer (TA-XT2i of Stable Micro Systems, Godalming, UK), and a TPA (texture profile analysis) test was carried out using chicken muscle samples of cylindrical shape. For this test, a non-lubricated flat platform, a 60-mm cylindrical compression probe and a 25 kg load cell were used. The samples were twice compressed using a fixed rate (1 mm/s) at 50% deformation. Amongst the different parameters calculated, elasticity was found to be the more representative. All measurements were performed in triplicate.

### 3.5. Statistical Analysis

The statistical analysis of the results was performed by analysis of variance (ANOVA) at a 95% significance level using STATISTICA^®^ 7.0 (StatSoft Inc., Tulsa, OK, USA). Duncan’s multiple range test was used for the evaluation of significant differences (a = 0.05).

## 4. Conclusions

The objective of the present study was to study the benefits of applying an osmotic dehydration step, with and without a prior step of bioactive compounds impregnation into chicken fillet muscle. Osmotic dehydration procedure as a sole technique caused a significant water activity decrease (down to 0.91) and improved quality stability during subsequent chilled storage in terms of both microbial decay and rancidity development. On the other hand, the enrichment of chicken samples with antimicrobial/antioxidant compounds after dipping in an oregano infusion seemed to have a more pronounced effect on lipid oxidation inhibition compared to microbial growth of spoilage bacteria. In order to assess the shelf life of refrigerated chicken flesh, criteria based on total microflora growth and TBARS increase were applied, the latter being directly associated with sensory perception of undesirable odors. As a concluding remark, the combined treatment of impregnation with a phenolic-rich oregano infusion followed by a water activity lowering step (osmotic dehydration) can be considered the most effective technique according to hurdle technology principles for the preservation of chicken fillets. In fact, one of the main findings of this work is that it might be possible to apply a mild a_w_-decreasing treatment (e.g., osmotic dehydration) as an effective alternative to partially «substitute» the antimicrobial action of polyphenols in essential oils, avoiding the unpleasant modification of sensory attributes that is frequently caused; at the same time, this research confirmed the importance of water-soluble polyphenols as a significant antioxidant factor. In any case, a thorough and systematic sensory analysis should be simultaneously performed in order to optimize impregnation conditions (oregano concentration, time/temperature of immersion), aiming at obtaining a novel, stable and organoleptically superior chicken fillet product with extended shelf life under chilled storage.

## Figures and Tables

**Table 1:** Growth rates and final population of the total viable count. Psychrotrophic and LAB of chicken fillet samples stored at 4 °C (mean values ± standard error based on the statistical variation in the kinetic parameters of the Baranyi growth model—regression analysis).

	Total Viable Count			Psychrotrophic Bacteria			LAB		
	k (d^−1^)	N_o_	N_max_	k (d^−1^)	N_o_	N_max_	k (d^−1^)	N_o_	N_max_
Untreated	0.65 ± 0.17 ^a^	6.6 ± 0.3 ^a^	10.2 ± 0.2 ^a^	0.17 ± 0.02 ^a^	5.9 ± 0.4 ^a^	9.8 ± 0.3 ^a^	0.38 ± 0.08 ^a^	3.9 ± 0.4 ^a^	8.3 ± 0.5 ^a^
OD	0.17 ± 0.08 ^b^	4.8 ± 0.4 ^b^	7.8 ± 0.5 ^b^	0.10 ± 0.06 ^b^	3.7 ± 0.4 ^b^	7.3 ± 0.3 ^b^	0.23 ± 0.07 ^b^	3.0 ± 0.5 ^b^	7.2 ± 0.6 ^b^
OV/OD	0.19 ± 0.09 ^b^	4.5 ± 0.1 ^b^	8.0 ± 0.1 ^b^	0.08 ± 0.05 ^b^	3.7 ± 0.1 ^b^	6.8 ± 0.2 ^b^	0.21 ± 0.05 ^c^	2.9 ± 0.3 ^b^	7.4 ± 0.2 ^b^

^a,b^ Different superscripts in the same column indicate significant differences (*p* < 0.05).

## Data Availability

Not applicable.

## Sample Availability

Samples of the materials (oregano infusion and osmotic solutions) and samples of treated chicken fillets are available from the authors.
